# Evaluating the efficacy of low-molecular-weight heparin in managing umbilical artery thrombosis during pregnancy: does it offer therapeutic benefits?

**DOI:** 10.3389/fmed.2025.1540685

**Published:** 2025-03-24

**Authors:** Peng Zhao, Yicheng Lu, Sitong Liu, Lidan Zhang, Chong Chen, Xiaofu Yang

**Affiliations:** ^1^Department of Obstetrics, Women’s Hospital, Zhejiang University School of Medicine, Hangzhou, China; ^2^Department of Ultrasonography, Women’s Hospital, Zhejiang University School of Medicine, Hangzhou, China

**Keywords:** umbilical artery thrombosis, expectant management, low molecular weight heparin, birth weight, anti-coagulation therapy

## Abstract

**Introduction:**

Umbilical artery thrombosis (UAT) is a rare but serious pregnancy complication, potentially causing fetal growth restriction, distress, and stillbirth. Diagnosis relies on Doppler ultrasound and pathological assessment. Close monitoring and potential low-molecular-weight heparin (LMWH) therapy aim to prolong gestation and improve outcomes, but debate persists on its efficacy compared to expectant management.

**Methods:**

A retrospective study, conducted between January 2013 and December 2023, enrolled singleton pregnant women diagnosed with UAT during pregnancy. The experiment group included pregnant women who underwent LMWH with anti-coagulation therapy during pregnancy, while the expectant group comprised pregnancies that received standard prenatal care without any specific intervention for UAT.

**Results:**

The expectant group showed a significant increase in birth weight (expectant vs. experiment: 2434.40 ± 770.20 g vs. 1874.46 ± 717.83 g, *P* < 0.05) and a significant decrease in the incidence of births before 34 weeks (expectant vs. experiment: 42.24% vs. 82.75%, *P* < 0.05). Gestational age at birth was notably higher in the expectant group as compared to the experiment group (35.32 ± 3.89 vs. 33.59 ± 4.17), although the difference did not reach statistical significance (*p* = 0.110). The multi-factor ANOVA revealed statistically significant effects of anti-coagulation therapy (*F* = 4.479, *p* = 0.039) and gestational age at birth (*F* = 179.110, *p* = 0.000) on birth weight. This study found that the relationship between these variables can be formulated as: birth weight = −3314.782–256.106 × anti-coagulation therapy (coded as 1 if yes and 0 if no) +161.858 × gestational age at birth.

**Conclusion:**

Our study suggests that expectant therapy may offer substantial benefits compared to experimental therapy involving the administration of LMWH.

## Introduction

Umbilical artery thrombosis (UAT), a rare pregnancy complication, has an estimated incidence ranging from 0.0025 to 0.045% (1). This condition can lead to fetal growth restriction, fetal distress, and stillbirth (2, 3). Currently, the diagnosis of UAT relies primarily on Doppler ultrasound imaging and pathological assessment of the umbilical cord after delivery.

UAT carries the risk of sudden fetal death, deciding to terminate pregnancy a potentially viable option in the third trimester to avoid intrauterine fetal death; however, it is imperative to acknowledge that this course of action may result in an increased risk of iatrogenic preterm birth. Therefore, close monitoring of fetal conditions and growth trends via therapeutic management has been recognized as a strategy that potentially prolongs the gestational period and enhances neonatal outcomes (2, 4, 5). For instance, Wang et al. reported that the administration of low-molecular-weight heparin (LMWH) to prevent the progression of UAT holds the potential to enhance pregnancy outcomes (6). Additionally, Li noted that anticoagulation therapy of LMWH combined with aspirin could reduce the occurrence of adverse pregnancy outcomes (7). However, in other research on expectant management where the application of LMWH was not used, similar perinatal outcomes were achieved (3, 5, 8, 9). The question of whether LMWH holds a distinct advantage over expectant management remains a subject of ongoing debate, particularly in terms of its potential therapeutic benefits. Consequently, we conducted the current study to evaluate the efficacy of LMWH in managing UAT during pregnancy.

## Methods

This study was conducted retrospectively, involving a comprehensive review of all delivery cases recorded at the Women’s Hospital, Zhejiang University School of Medicine, between January 2013 and December 2023. Patients suspected of having UAT, based on either ultrasonographic imaging or pathological examination, underwent a comprehensive review by ultrasonography experts and pathology experts, respectively. Following this thorough evaluation, only those participants with a confirmed diagnosis of UAT were selected and included in the study.

### Ultrasonographic imaging

The ultrasound screening is performed by the direct visualization of the umbilical cord or by tracking the umbilical arteries around the fetal bladder with color Doppler technology (10). The diagnosis of UAT is established when an initial ultrasound examination in the first trimester of pregnancy indicates normal umbilical artery flow, but later scans reveal the presence of a single umbilical artery.

### Pathological examination

Upon staining with Hematoxylin–Eosin, sections of the umbilical cord revealed the presence of thrombosis (specifically, fibrinous, mixed, or red thrombus) within one of the umbilical arteries. This thrombosis exhibited features that ranged from total or partial necrosis of the artery wall to cases where no evident necrosis was observed (11).

### Group assignment

The study population was assigned into two distinct groups: the experimental group and the expectant group, based on the treatment regimen they received. Participants in the experimental group received LMWH (nadroparin calcium 4,100 U daily or enoxaparin sodium 4,000 U daily) for anti-coagulation therapy, underwent rigorous ultrasound surveillance, non-stress testing beyond 28 weeks of gestation, and were instructed to closely monitor fetal movements. In contrast, participants in the expectant group received only standard prenatal care.

### Data collection

Data pertaining to maternal age, gravidity, parity, gestational weeks at diagnosis, gestational weeks at delivery, and neonatal outcomes such as birth weight, Apgar scores, cesarean delivery, fetal distress, neonatal morbidity, and newborn intensive care unit (NICU) admission were collected and analyzed.

### Statistical analysis

Statistical analysis was carried out with SPSS 25.0 for Microsoft Windows (IBM Corp., Armonk, NY, USA). Comparisons of continuous variables between groups were performed using Student’s t-test (for data that were normally distributed) or Mann–Whitney U-test (for data that exhibited non-normal distribution), while comparisons of categorical variables were conducted with the χ^2^ test. Multi-factor ANOVA was used to investigate the effects of independent variables on birth weight. Multivariable logistic regression models were utilized to explore the association between the independent variables and birth weight. A *p*-value of < 0.05 was considered statistically significant.

## Results

### Participants selection

**Figure 1:**
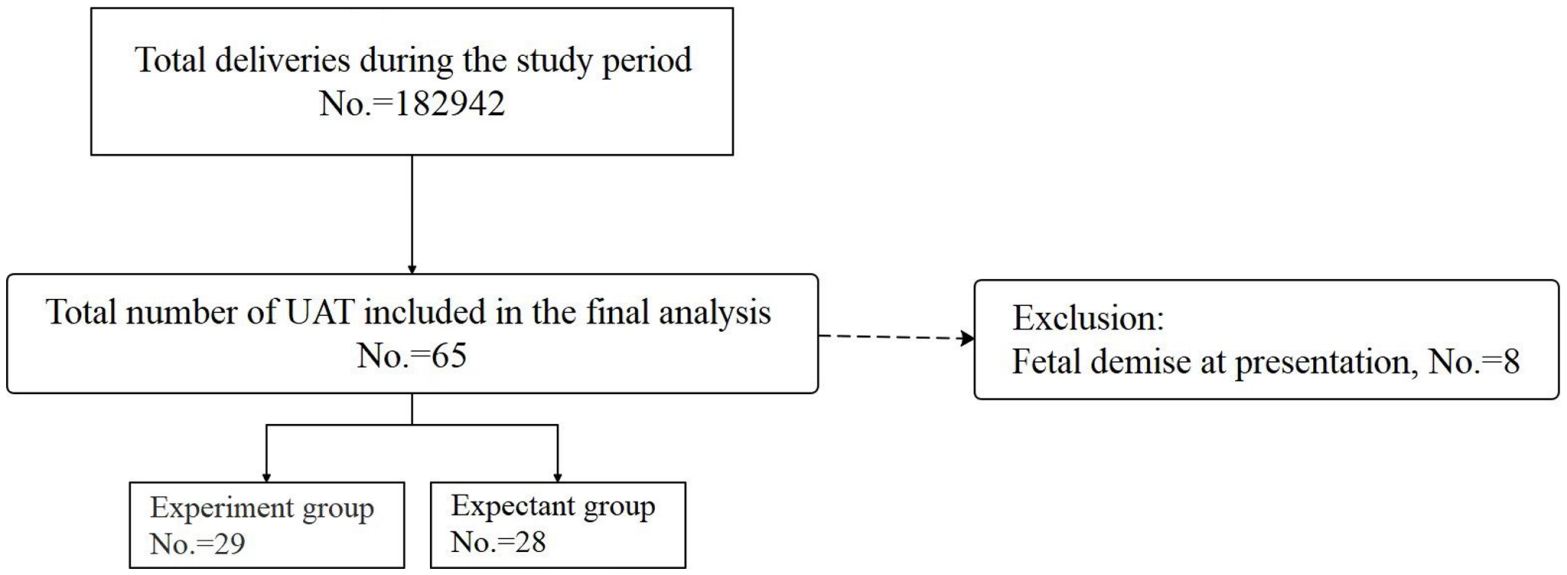
Flow diagram.

During the study period, a total of 182,942 deliveries were recorded. Among these, 65 pregnancies were diagnosed with UAT. However, eight cases were subsequently excluded due to fetal death at presentation. Finally, a total of 57 participants with UAT were included in the analysis, with 29 participants assigned to the experiment group and the remaining 28 designated to the expectant group. Details are presented in Figure 1.

### Clinical characteristics

The results of the comparison pertaining to baseline clinical characteristics between the experiment group and the expectant group are presented in Table 1. Consequently, no significant differences were noted in terms of maternal age (30.14 ± 4.23 vs. 30.79 ± 3.92, *p* = 0.552), BMI (26.75 ± 4.99 vs. 26.05 ± 3.09, *p* = 0.527), gravidity (1.97 ± 1.38 vs. 2.07 ± 1.33, *p* = 0.769), primigravida (48.28% vs. 46.43%, *p* = 1.000), parity (0.41 ± 0.68 vs. 0.43 ± 0.57, *p* = 0.930), primipara (31.03% vs. 39.29%, *p* = 0.585), and gestational age at diagnosis (29.90 ± 4.62 vs. 31.00 ± 4.31, *p* = 0.497).

**Table 1:** Comparison of clinical characteristics between the experiment group and expectant group.

	Experiment group *N* = 29	Expectant group *N* = 28	*p*-value
Maternal age	30.14 ± 4.23	30.79 ± 3.92	0.552
BMI	26.75 ± 4.99	26.05 ± 3.09	0.527
Gravidity	1.97 ± 1.38	2.07 ± 1.33	0.769
Primigravida	14 (48.28)	13 (46.43)	1.000
Multigravida	15 (51.72)	15 (53.57)	
Parity	0.41 ± 0.68	0.43 ± 0.57	0.930
Primipara	20 (68.97)	17 (60.71)	0.585
Multipara	9 (31.03)	11 (39.29)	
Gestational age at diagnosis (weeks)	29.90 ± 4.62	31.00 ± 4.31	0.497

### Clinical outcomes

The results of the comparison of clinical outcomes between the experiment group and the expectant group are presented in Table 2. The birth weight in the experiment group was significantly lower compared to the expectant group (1874.46 ± 717.83 vs. 2434.40 ± 770.20, *p* = 0.008), while the proportion of births before 34 weeks was significantly higher in the experiment group than the expectant group (82.75% vs. 42.24%, *p* = 0.001), indicating a higher prevalence of early preterm delivery (<34 weeks of gestation) and lower birth weight in the experiment group. Gestational age at birth was notably lower in the experiment group as compared to the expectant group, although the difference did not reach statistical significance. No significant differences were noted in terms of Apgar scores, cesarean delivery, fetal distress, neonatal morbidity, and NICU stay.

**Table 2:** Perinatal outcomes of the experiment group and expectant group.

Outcomes	Experiment group *N* = 29	Expectant group *N* = 28	*p*-value
<34 weeks	24 (82.75)	11 (42.24)	0.001
<32 weeks	17 (58.62)	9 (32.14)	0.064
<30 weeks	13 (44.82)	6 (21.43)	0.092
<28 weeks	5 (17.24)	3 (10.71)	0.706
Gestational age at birth (weeks)	33.59 ± 4.17	35.32 ± 3.89	0.110
Birth weight	1874.46 ± 717.83	2434.40 ± 770.20	0.008
Apgar score 1 min	8.54 ± 2.47	9.40 ± 1.29	0.125
Apgar score 5 min	9.92 ± 0.41	9.96 ± 0.20	0.618
Cesarean delivery	25 (86.21)	24 (85.71)	1.000
Fetal distress	10 (34.48)	11 (39.29)	0.707
Neonatal morbidity	3 (10.34)	3 (10.71)	1.000
NICU stay (days)	20.24 ± 22.88	9.52 ± 16.80	0.070

Data are presented as mean ± SD or n (%).

### Multi-factor ANOVA

The results of the multi-factor ANOVA are summarized in Table 3. The ANOVA revealed statistically significant anti-coagulation therapy (LMWH for participants in the experiment group) (*F* = 4.479, *p* = 0.039) and gestational age at birth (*F* = 179.110, *p* = 0.000) on birth weight.

**Table 3:** Multi-factor ANOVA for birth weight: anti-coagulation therapy, gestational age at birth, and gender.

Outcome	*F*	*p*
Experiment therapy (anti-coagulation therapy)	4.479	0.039
Gestational age at birth (weeks)	179.110	0.000
Gender	1.917	0.172

### Linear regression model

The results in Table 4 indicated a significant negative association of anti-coagulation therapy (*β* = −0.164, *p* = 0.014) and a strong positive correlation of gestational age (β = 0.846, *p* < 0.001) with birth weight. The final linear regression equation can be expressed as: birth weight = −3314.782–256.106 × anti-coagulation therapy +161.858 × gestational age at birth (weeks).

**Table 4:** Linear regression model for experiment therapy and gestational age at birth (weeks).

Items	Non-standardized Coefficients		Standardized Coefficients			95% Confidence interval for B	
	Regression coefficient B	Std.-Error	Beta	*T*	Sig.	Lower limit	Upper limit
(Constant value)	−3314.782	441.554		−7.507	0.000	−4201.669	−2427.894
Experiment therapy (Anti-coagulation therapy)	−256.106	100.2	−0.164	−2.555	0.014	−457.410	−54.802
Gestational age at birth (weeks)	161.858	12.270	0.846	13.191	0.000	137.213	186.502

## Discussion

### Major findings

**Figure 2:**
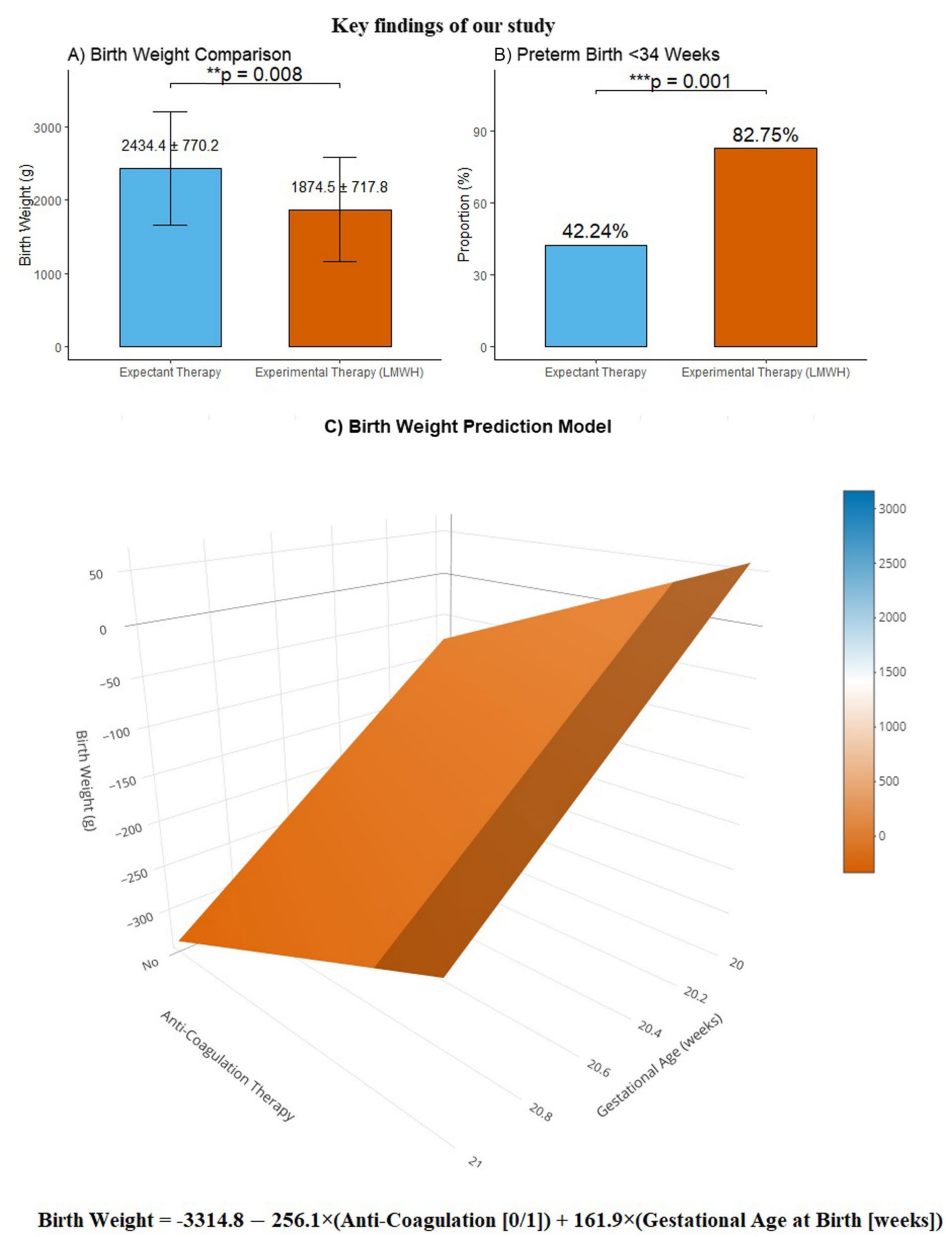
The key findings of our study. Panel **(A)** presents a comparative analysis of birth weight, demonstrating that the birth weight in the experiment group was significantly lower compared to the expectant group (1874.46 ± 717.83 vs. 2434.40 ± 770.20, *p* = 0.008). Panel **(B)** illustrates the distribution of preterm births before 34 weeks, showing that the proportion of births before 34 weeks was significantly higher in the experiment group than the expectant group (82.75% vs. 42.24%, *p* = 0.001). Panel **(C)** displays our birth weight prediction model, featuring the mathematical formula: “birth weight = −3314.782–256.106 × anti-coagulation therapy +161.858 × gestational age at birth”, presented in a three-dimensional surface plot for enhanced visualization.

The key findings of our study were: (1) expectant therapy had a significant advantage over experiment therapy in pregnancies with UAT, as the administration of LMWH resulted in a significant decrease in birth weight and a substantial increase in the incidence of preterm birth (<34 weeks); (2) the relationship between these variables can be formulated as: birth weight = −3314.782–256.106 × anti-coagulation therapy +161.858 × gestational age at birth. The key findings are incorporated and presented in Figure 2.

### Comparisons with existing literature

UAT may occur after placenta thrombotic vasculopathy (12), umbilical cord abnormalities (3, 13, 14), and underlying maternal diseases (2, 15). At present, no consensus has been reached on the treatment strategy for UAT. In clinical practice, upon the occurrence of a fetal umbilical cord embolism, one would instinctively consider the implementation of anti-coagulation therapy to arrest the progression of thrombus emboli, thereby preventing complete occlusion of the umbilical vessels and reducing the subsequent risk of fetal death. For instance, Wang et al. (6) reviewed 10 cases of pregnancies with UAT. Notably, all participants in the study received treatment with LMWH, and there was no control group of expectant mothers. Based on their findings, the researchers concluded that the early administration of LMWHs may enhance pregnancy outcomes. However, there have been arguments raised against the use of anticoagulation treatment. Wei et al. reported a case series revealing that the expectant management of UAT had comparable fetal outcomes to those observed in patients who received anti-coagulation management (3). Han et al. demonstrated that expectant treatment of patients with UAT had apparent positive effects for extending gestational age, which was supported by another study conducted by Dindinger et al. (5, 9). Our study found that expectant management had significant advantages compared to anti-coagulation therapy, as the latter, when combined with frequent ultrasound surveillance, could induce anxiety in both patients and clinicians, resulting in unnecessary early medical interventions to deliver the fetus.

### Strengths and limitations

Our study has several strengths. First, it was designed as a retrospective study with two arms (expectant vs. experiment), whereas the previous studies (2–4, 6, 7) were primarily case reports. Second, we used multi-factor ANOVA to analyze the associations between the expectant group and the experiment group, leveraging its advantages in accounting for the potential confounding effects of multiple variables. Third, due to the rigorous design and comprehensive analysis, our results were more aligned with real-world clinical logic and practical experiences, indicating a greater degree of applicability and reliability. However, it is important to acknowledge that the retrospective design of our study, coupled with the relatively small sample size, may result in incomplete data and an increased risk of bias.

## Conclusion

In conclusion, our study provides valuable insights into the management of UAT and suggests that expectant therapy may offer significant advantages over experimental therapy involving LMWH administration. These findings have important implications for clinical practice and future research in this area.

## Data Availability

The original contributions presented in the study are included in the article/supplementary material, further inquiries can be directed to the corresponding author/s.
